# Sex-specific associations between blood urea nitrogen and risk of hyperuricemia in U.S. adults: the NHANES 1999-2020

**DOI:** 10.3389/fendo.2025.1560738

**Published:** 2025-06-05

**Authors:** Lingling Chen, Lixue Yin

**Affiliations:** ^1^ Ultrasound in Cardiac Electrophysiology and Biomechanics Key Laboratory of Sichuan Province, Sichuan Provincial People’s Hospital, University of Electronic Science and Technology of China, Chengdu, China; ^2^ Department of Cardiovascular Ultrasound & Noninvasive Cardiology, Sichuan Provincial People’s Hospital, University of Electronic Science and Technology of China, Chengdu, China

**Keywords:** hyperuricemia, blood urea nitrogen, risk, gender medicine, NHANES

## Abstract

**Background:**

Blood urea nitrogen (BUN), one of the recognized indicators of renal function, is a key marker of metabolic diseases, but there are few data on the association of BUN levels with hyperuricemia (HUA) in the general adult population. The aim of the study is to explore the relationship between BUN and HUA in the general population and the potential impact of gender on this relationship.

**Methods:**

This study was conducted involving 17,846 adults from the National Health and Nutrition Examination Survey (NHANES) between 1999-2020. Data on age, gender, race, marital status, education level, height, weight, body mass index (BMI), waist circumference (WC), systolic blood pressure (SBP), diastolic blood pressure (DBP), triglyceride (TG), total cholesterol (TC), high-density lipoprotein cholesterol (HDL-C), low-density lipoprotein cholesterol (LDL-C), fasting plasma glucose (FPG), hemoglobin A1c (HbA1C), serum uric acid (SUA), BUN, creatinine, and albumin were collected from all participants. Multivariate logistic regression, curve fitting and subgroup analyses were employed to investigate the associations between BUN and HUA stratified by sex.

**Results:**

After weighted analysis, the results of this study represented approximately 164.42 million U.S. adults. The overall prevalence of HUA was 18.22%, and 20.72% in males and 15.82% in females. In the fully adjusted model, there was a positive association between BUN and HUA and this positive association remained significantly stratified by sex. Smoothed curve-fitting analysis revealed that the dose-response relationship between BUN and the risk of developing HUA was linear in men and nonlinear in women. There was evidence of an interaction between BUN levels and gender status that increased the risk of HUA and the OR for the association between BUN and HUA was higher in females than in males. Subgroup analyses showed that the association between BUN and the risk of developing HUA remained consistently positive across all subgroups in both male and female participants.

**Conclusions:**

This study confirmed that BUN were positively associated with HUA among U.S. adults that remained significant when stratified by sex, but there were gender differences in the form and extent of this positive correlation.

## Introduction

Hyperuricemia (HUA), a metabolic disorder characterized by dysregulated uric acid homeostasis involving either overproduction or impaired excretion, has been pathophysiologically linked to gout pathogenesis and chronic kidney disease progression (1). Contemporary epidemiological studies have established significant associations between HUA and other metabolic disorders such as obesity, hypertension, dyslipidemia, and nonalcoholic fatty liver disease (2–7). The etiology of HUA is multifactorial, involving a complex interplay of genetic predisposition and environmental influences. Unhealthy lifestyle habits, such as poor diet and sedentary behavior, are known to exacerbate the risk of developing HUA (8, 9). HUA can affect patients of all ages and genders, and its prevalence is on the rise globally; as of 2016, the global prevalence of HUA had reached 21%, with its prevalence varying by geographic region. Nationally representative surveys revealed concerning temporal trends: NHANES 2015–2016 documented 20% prevalence among U.S. adults (10), while Chinese adult populations demonstrated accelerated prevalence increases from 11.1% (2015-16) to 14.0% (2018-19) (11). Importantly, longitudinal cohort studies have shown that individuals with HUA are at a higher risk of mortality (12, 13). Therefore, gaining a comprehensive understanding of HUA and its associated factors is crucial for effective prevention and management strategies, ultimately improving the prognosis and quality of life for individuals with HUA. BUN serves as a clinically validated biomarker of renal filtration efficiency, quantitatively reflecting circulating urea concentrations derived from exogenous protein catabolism and endogenous nitrogen homeostasis. Its serum levels are physiologically modulated by multifactorial determinants including dietary protein intake, proteolytic metabolic pathways, hydration homeostasis, hepatic urea cycle enzymatic activity, and renal excretory capacity, thereby constituting a critical diagnostic parameter for both nephrological evaluation and nutritional status assessment (14). As research related to BUN has intensified, many studies have found that high levels of BUN are associated with a poor prognosis in a number of acute or critical illnesses, including severe cardiovascular and cerebrovascular events [e.g., acute coronary syndromes (15), acute aortic coarctation (16), cardiogenic shock (17), and acute ischemic strokes (18)], neonatal sepsis (19), critical limb ischemia, acute exacerbation of chronic obstructive pulmonary disease (20), and primary pulmonary hypertension (21). Recently, there has been interest and attempts to investigate the relationship between BUN and metabolic diseases [including diabetes mellitus (DM) (22), hyperlipidemia (23), hypertension and fatty liver (24, 25)], as well as the potential role of BUN in the diagnosis and treatment of metabolic diseases. Notably, a critical knowledge gap persists regarding BUN-HUA interrelationships within general adult populations, particularly in the United States. Therefore, this study aims to explore the relationship between BUN and HUA in the general population, as well as the potential impact of gender on this relationship.

## Materials and methods

### Study population

The National Health and Nutrition Examination Survey (NHANES) is an open comprehensive program of research studies aimed at evaluating the health and nutritional status of both adults and children across the United States. This initiative is overseen by the Centers for Disease Control and Prevention (CDC). What sets NHANES apart is its distinctive approach, which integrates both personal interviews and thorough physical examinations. Each participant provided written informed consent, and all procedures were sanctioned by the NCHS Research Ethics Review Board.

**Figure 1:**
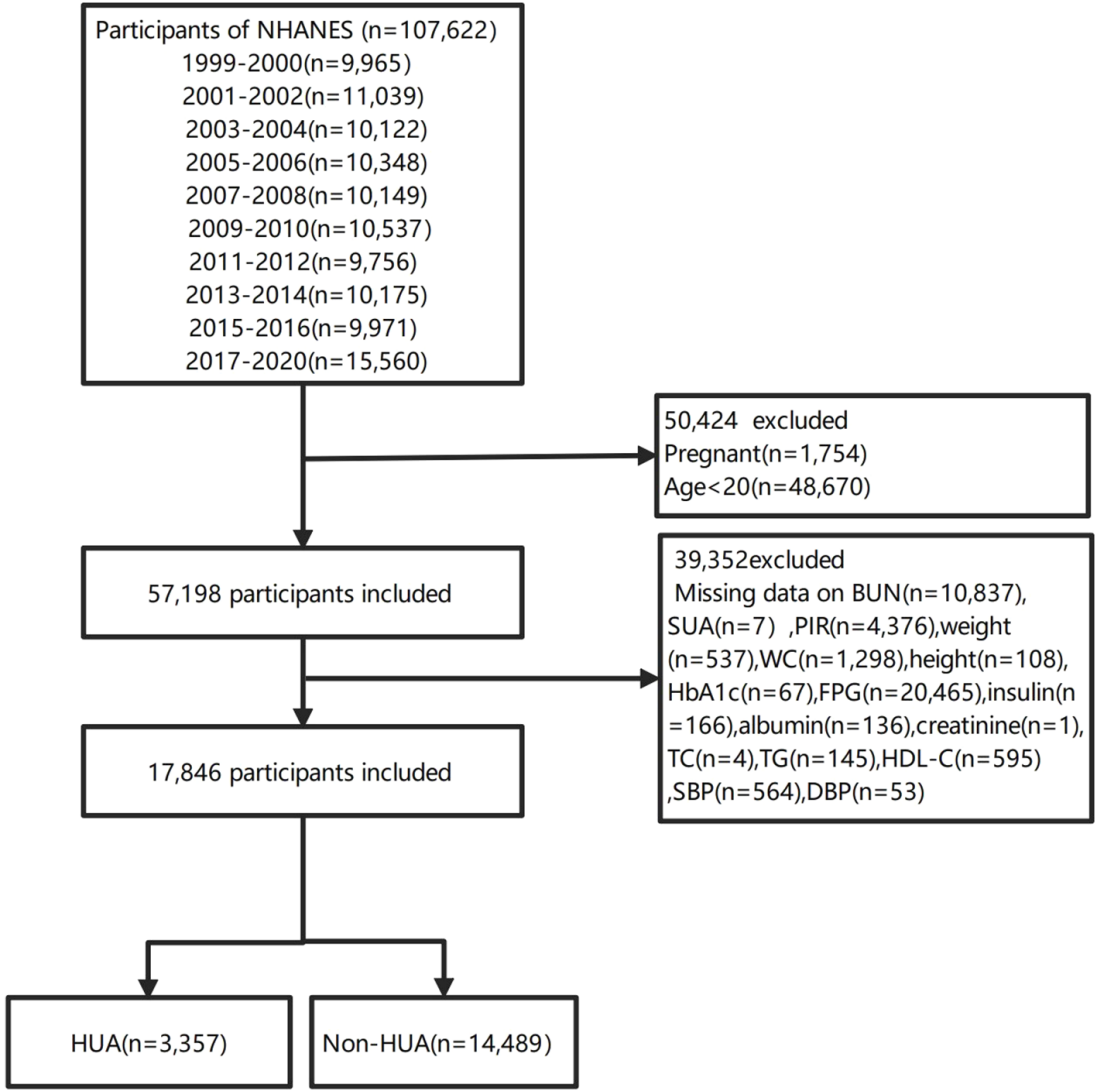
Flowchart of participants selection.

In our investigation, we compiled data from ten NHANES cycles from 1999 to 2020.3, which encompassed a total of 107,622 individuals. To maintain the focus of our study, we omitted pregnant women (n=1754), individuals under the age of 20(n=48670). To ensure the precision and uniformity of our data, we further excluded participants with missing BUN(n=10837), SUA(n=7), family poverty income ratio (PIR) data (n=4376), weight(n=537), WC(n=1298), height(n=108), HbA1c(n=67), FPG(n=20465), insulin(n=166), albumin(n=136), creatinine(n=1), TC(n=4), TG(n=145), HDL-C(n=595), SBP(n=564) and DBP(n=53). Consequently, our analysis was conducted on a cohort of 17,846 participants, comprising 3,357 with HUA and 14,489 without HUA (non-HUA). The participant enrollment flowchart is depicted in **Figure 1**.

### Definition of HUA

HUA is characterized by overproduction or under-excretion of uric acid. In this study, HUA was quantified using SUA measurements from the NHANES data, defined as 420umol/L (7.0 mg/dl) for men and 360umol/L (6.0 mg/dl) for women (26).

### Covariates

The sociodemographic covariates included age, sex, race, marital status, and education level. Health-related covariates included height, weight, WC, BMI, SBP, DBP, as well as smoking and drinking habits. Venous blood samples were obtained to determine the serum concentrations of TG, TC, HDL-C, LDL-C, FPG, HbA1c, SUA, BUN, creatinine, and albumin.

A range of potential covariates, including age, sex, race, marital status, education level, family income, smoking and drinking habits, hypertension, hyperlipidemia and DM, were evaluated on the basis of literature. Age was categorized according to the World Health Organization (WHO) into four groups: young individuals (≤44 years), middle-aged individuals (45–59 years), younger elderly (60–74 years), and older elderly (75–89 years).The race categories included Mexican American, other Hispanic, non-Hispanic White, non-Hispanic Black, and other races. Marital status was categorized as married/living with a partner, widowed/divorced/separated, or never married. Educational attainment was divided into three groups: less than high school, high school diploma (including GED), and more than high school. A US government report classified family income into low-income (PIR ≤ 1.3), middle-income (PIR>1.3–3.5), and high-income (PIR≥3.5) (27). Individuals who answered “yes” to the question “Have you smoked at least 100 cigarettes in your entire life?” were classified as “yes” in terms of smoking status, and subjects who answered “no” or refused to answer the question were categorized as “no” smoking status. Individuals who answered “Yes” to the question “Had you had at least 12 alcohol drinks/1 yr?” were classified as “Yes” of drinking status, and subjects who answered “no” or refused to answer the question were categorized as “No” drinking status.

The outcome of hypertension was defined as a mean SBP of ≥130 mmHg, a mean DBP of ≥80 mmHg, a self-reported hypertension diagnosis, or the use of an antihypertensive drug (28). Adult Treatment Panel III (ATP 3) of the National Cholesterol Education Program (NCEP) classified hyperlipidemia as TC≥200 mg/dL, TG≥150 mg/dL, HDL-C (40<mg/dL in men and <50mg/dL in women) or LDL-C≥130 mg/dL (29). Alternately, persons who reported using cholesterol-lowering drugs were also classified as having hyperlipidemia. According to the ADA’s DM diagnostic criteria, DM was defined by self-reported diagnosis, the use of insulin or oral hypoglycemic medication, a FPG ≥ 126 mg/dL or an HbA1c level ≥ 6.5% (30).

### Statistical analysis

The study was a secondary analysis of publicly available datasets. We used weights for the weighted analysis. For the combined analyses of NHANES 1999–2000 and 2001–2002.3 data, a four-year fasting subsample weight (WTSAF4YR) set was used; for 2003–2004, 2005–2006, 2007–2008, 2009–2010, 2011–2012, 2013–2014, and 2015–2016, a two-year fasting subsample weight (WTSAF2YR) set was used; and for 2017–2020.3, the special fasting subsample weight (WTSAFPRP) set was used. The sampling weights for 1999–2020.3 were calculated as follows:1999–2000 and 2001–2002 weights were WTSAF4YRx2/10.625, 2003–2004,2005–2006,2007–2008, 2009–2010, 2011–2012, 2013–2014, and 2015–2016 weights were WTSAF2YRx1/10.625, and the 2017–2020.3 weight was WTSAFPx1.625/10.625.

The characteristics of the participants were described as the means (standard deviations, SDs) for continuous variables and numbers and percentage frequencies (%) for categorical variables. Comparison of continuous variables among groups was performed with the use of the independent samples Student’s t-test or Mann-Whitney U-test depending on the normality of the distribution, and categorical data were compared by chi-square or Fisher’s exact test as appropriate.

BUN was categorized into three quantiles (Q1-Q3) and calculated the *p* for trend in order to verify the results of BUN as the continuous variable, and to examine the possibility of nonlinearity. Multivariate logistic regression analyses were performed via four models to examine the associations between BUN and HUA, with the lowest quantile used as the reference category, and the results were presented as ORs with 95% CIs. On the basis of clinical judgment and previous scientific literature, four models were constructed as follows: Model 1: unadjusted; Model 2: age, race, gender, marital status and education level; Model 3, adjusted for age, race, gender, marital status, education level, WC, BMI, insulin, albumin and creatinine; Model 4, adjusted for age, race, gender, marital status, education level, WC, BMI, insulin, albumin, hypertension status, DM status, smoking status, drinking status and hyperlipidemia status.

A generalized additive model (GAM) and restricted cubic spine (RCS) model were used to examine the possible nonlinear dose-response associations between BUN and HUA by sex. Besides, we performed interaction and subgroup analyses according to age group, race, marital status, education level, smoking status, drinking status, hypertension status, hyperlipidemia status and DM status via logistic regression models by sex.

Participants’ BUN levels and risk of HUA were compared among male and female subjects. The multivariate logistic regression model was used to perform subgroup analysis based on different gender. Interactions between subgroups were examined by likelihood ratio testing.

Statistical testing was two-sided with a level of significance set at *p* = 0.05. All analyses were performed with the Free Statistics platform (Version 1.9, Beijing, China, http://www.clinicalscientists.cn/freestatistics) and the statistical software packages R (http://www.R-project.org, The R Foundation).

## Results

### Baseline characteristics of the participants

Among all participants,3357(18.8%) participants were diagnosed with HUA, and14489(81.2%) did not have HUA (**Figure 1**). This study included 17,846 participants with available data for analysis are shown in **Tables 1** and **2**, representing approximately 164.42 million US adults aged ≥20 years after weighted analysis. The baseline characteristics of the participants were presented by BUN quantiles as follows: Q1 ≤ 3.93 mmol/L; Q2: 3.93~5.36 mmol/L; Q3≥5.36 mmol/L. The mean (SD) BUN was 4.853 mmol/L (SD:1.854). The weighted mean age was 46.900 ± 16.645 years, and 51.04% of the participants were female.

**Table 1:** Baseline characteristics of participants by BUN quantile.

Variables	BUN quantile				*p* value	SMD
	Overall (n=164420915)	Q1 (n=45045606.91)	Q2 (n=60869278.91)	Q3 (n=58506029.14)		
age (mean (SD)) (years)	46.900 (16.645)	40.248 (14.062)	45.059 (15.684)	53.936 (16.834)	<0.0001	0.5837
Race, n (%)						
Mexican American	12722453.81 (7.74)	4305831.63 (9.56)	4883036.63 (8.02)	3533585.55 (6.04)	<0.0001	0.2389
Other Hispanic	9153799.71 (5.57)	2571651.66 (5.71)	3656482.93 (6.01)	2925665.11 (5.00)		
Non-Hispanic White	113809387.53 (69.22)	27812535.06 (61.74)	41652046.90 (68.43)	44344805.57 (75.80)		
Non-Hispanic Black	17325150.69 (10.54)	7182146.06 (15.94)	6212210.60 (10.21)	3930794.03 (6.72)		
Other Race	11410123.22 (6.94)	3173442.49 (7.04)	4465501.85 (7.34)	3771178.87 (6.45)		
Gender, n (%)						
Male	80506454.23 (48.96)	15284100.92 (33.93)	30455548.63 (50.03)	34766804.68 (59.42)	<0.0001	0.3496
Female	83914460.73 (51.04)	29761505.99 (66.07)	30413730.28 (49.97)	23739224.45 (40.58)		
Marital status, n (%)						
Married/Living with Partner	106040171.64 (64.49)	26458491.97 (58.74)	39846984.85 (65.46)	39734694.82 (67.92)	<0.0001	0.232
Widowed/Divorced/Separated	33892379.19 (20.61)	8830283.30 (19.60)	11826162.46 (19.43)	13235933.44 (22.62)		
Never married	24488364.13 (14.89)	9756831.64 (21.66)	9196131.60 (15.11)	5535400.89 (9.46)		
Education level, n (%)						
Less Than High School	26165719.26 (15.91)	7975847.67 (17.71)	9136551.47 (15.01)	9053320.11 (15.47)	0.0173	0.0576
High School Diploma (including GED)	38925639.57 (23.67)	10842687.32 (24.07)	14080631.96 (23.13)	14002320.30 (23.93)		
More Than High School	99329556.13 (60.41)	26227071.92 (58.22)	37652095.48 (61.86)	35450388.73 (60.59)		
weight (mean (SD)) (kg)	82.471 (21.127)	79.383 (21.355)	83.259 (21.359)	84.030 (20.453)	<0.0001	0.1469
WC (mean (SD)) (cm)	98.442 (16.376)	95.729 (16.891)	98.488 (16.310)	100.484 (15.732)	<0.0001	0.1941
BMI (mean (SD)) (kg/m2)	28.775 (6.684)	28.318 (7.068)	28.896 (6.706)	29.002 (6.331)	0.0004	0.0674
height (mean (SD)) (cm)	169.063 (10.004)	167.266 (9.374)	169.513 (9.991)	169.977 (10.309)	<0.0001	0.1843
SBP (mean (SD))(mmHg)	120.675 (16.819)	118.036 (16.164)	120.008 (16.223)	123.401 (17.516)	<0.0001	0.2137
DBP (mean (SD)) (mmHg)	70.777 (12.017)	70.649 (11.867)	71.140 (11.503)	70.499 (12.633)	0.0644	0.0358
HbA1c (mean (SD)) (%)	5.566 (0.892)	5.444 (0.828)	5.536 (0.909)	5.692 (0.906)	<0.0001	0.1875
FPG (mean (SD)) (mmol/L)	5.819 (1.562)	5.569 (1.358)	5.785 (1.552)	6.048 (1.684)	<0.0001	0.2079
BUN (mean (SD)) (mmol/L)	4.853 (1.854)	3.050 (0.565)	4.458 (0.386)	6.652 (1.831)	<0.0001	2.4089
SUA (mean (SD) (umol/L)	324.783 (82.486)	298.752 (75.632)	322.437 (78.826)	347.267 (84.980)	<0.0001	0.4042
TC (mean (SD)) (mmol/L)	4.990 (1.040)	4.893 (1.040)	5.021 (1.006)	5.031 (1.068)	<0.0001	0.0884
LDL (mean (SD)) (mmol/L)	2.985 (0.914)	2.900 (0.920)	3.015 (0.877)	3.020 (0.943)	<0.0001	0.0873
HDL (mean (SD)) (mmol/L)	1.392 (0.411)	1.402 (0.416)	1.392 (0.409)	1.384 (0.408)	0.2952	0.0297
Smoking status, n (%)						
No	88909283.75 (54.07)	23703516.70 (52.62)	33550989.14 (55.12)	31654777.90 (54.11)	0.1657	0.0334
Yes	75511631.21 (45.93)	21342090.21 (47.38)	27318289.77 (44.88)	26851251.24 (45.89)		
Drinking status, n (%)						
No	121735863.01 (74.04)	34852107.14 (77.37)	45645191.72 (74.99)	41238564.15 (70.49)	<0.0001	0.1048
Yes	42685051.95 (25.96)	10193499.77 (22.63)	15224087.19 (25.01)	17267464.98 (29.51)		
Hypertension, n (%)						
No	90832431.39 (55.24)	26479130.31 (58.78)	34599904.51 (56.84)	29753396.57 (50.86)	<0.0001	0.1065
Yes	73588483.57 (44.76)	18566476.60 (41.22)	26269374.40 (43.16)	28752632.57 (49.14)		
Hyperlipidemia, n (%)						
No	44906784.78 (27.31)	14234251.17 (31.60)	17507895.42 (28.76)	13164638.20 (22.50)	<0.0001	0.1372
Yes	119514130.17 (72.69)	30811355.74 (68.40)	43361383.49 (71.24)	45341390.94 (77.50)		
DM, n (%)						
No	20590822.85 (12.52)	3625958.18 (8.05)	6514244.32 (10.70)	10450620.35 (17.86)	<0.0001	0.1974
Yes	143830092.10 (87.48)	41419648.73 (91.95)	54355034.59 (89.30)	48055408.79 (82.14)		
HUA, n (%)						
No	134462655.02 (81.78)	39355490.59 (87.37)	51127015.06 (83.99)	43980149.37 (75.17)	<0.0001	0.211
Yes	29958259.94 (18.22)	5690116.32 (12.63)	9742263.85 (16.01)	14525879.77 (24.83)		
PIR (median [IQR])	3.090 [1.560, 5.000]	2.590 [1.240, 4.450]	3.060 [1.570, 5.000]	3.490 [1.830, 5.000]	<0.0001	0.2217
TG (median [IQR]) (mmol/L)	1.150 [0.790, 1.680]	1.110 [0.768, 1.640]	1.152 [0.802, 1.671]	1.174 [0.813, 1.727]	0.0022	0.071
insulin (median [IQR]) (pmol/L)	55.140[36.000, 88.440]	53.144 [33.875, 85.740]	55.380[35.580, 89.918]	56.925 [37.260, 89.040]	0.0008	0.0497
albumin (median [IQR]) (mg/L)	7.500 [4.100, 14.500]	7.200 [3.800, 14.400]	7.300 [4.000, 13.200]	8.000 [4.400, 16.100]	<0.0001	0.0799
creatinine(median[IQR]) (umol/L)	10343.000 [6100.000, 15205.000]	9547.000 [5216.000, 15293.000]	10519.600[6188.000, 15204.800]	10784.800 [6718.400, 15293.000]	<0.0001	0.044

**Table 2:** Baseline characteristics of the participants according to sex.

Variables	Overall (n=164420915)	Male (n=80506454.23)	Female (n=83914460.73)	*p* value	SMD
Age (mean (SD))(years)	46.900 (16.645)	46.084 (16.471)	47.682 (16.773)	<0.0001	0.0962
Race, n (%)					
Mexican American	12722453.81 (7.74)	6831862.73 (8.49)	5890591.08 (7.02)	<0.0001	0.068
Other Hispanic	9153799.71 (5.57)	4354123.90 (5.41)	4799675.81 (5.72)		
Non-Hispanic White	113809387.53 (69.22)	55779849.47 (69.29)	58029538.06 (69.15)		
Non-Hispanic Black	17325150.69 (10.54)	7938669.41 (9.86)	9386481.28 (11.19)		
Other Race	11410123.22 (6.94)	5601948.73 (6.96)	5808174.49 (6.92)		
Marital status, n (%)					
Married/Living with Partner	106040171.64 (64.49)	54534762.72 (67.74)	51505408.92 (61.38)	<0.0001	0.249
Widowed/Divorced/Separated	33892379.19 (20.61)	12566429.78 (15.61)	21325949.41 (25.41)		
Never married	24488364.13 (14.89)	13405261.73 (16.65)	11083102.40 (13.21)		
Education level, n (%)					
Less Than High School	26165719.26 (15.91)	13694331.42 (17.01)	12471387.84 (14.86)	0.0004	0.0722
High School Diploma (including GED)	38925639.57 (23.67)	19521369.60 (24.25)	19404269.98 (23.12)		
More Than High School	99329556.13 (60.41)	47290753.22 (58.74)	52038802.91 (62.01)		
weight (mean (SD)) (kg)	82.471 (21.127)	88.939 (20.019)	76.266 (20.285)	<0.0001	0.6288
WC (mean (SD)) (cm)	98.442 (16.376)	101.063 (15.550)	95.928 (16.751)	<0.0001	0.3177
BMI (mean (SD)) (kg/m2)	28.775 (6.684)	28.584 (5.868)	28.959 (7.378)	0.0041	0.0564
height (mean (SD)) (cm)	169.063 (10.004)	176.185 (7.426)	162.230 (6.918)	<0.0001	1.9444
SBP(mean (SD)) (mmHg)	120.675 (16.819)	122.440 (15.148)	118.981 (18.118)	<0.0001	0.2071
DBP (mean (SD)) (mmHg)	70.777 (12.017)	72.119 (12.018)	69.491 (11.876)	<0.0001	0.22
HbA1c (mean (SD)) (%)	5.566 (0.892)	5.588 (0.933)	5.546 (0.851)	0.0018	0.0474
FPG (mean (SD)) (mmol/L)	5.819 (1.562)	5.971 (1.653)	5.674 (1.456)	<0.0001	0.1904
BUN (mean (SD)) (mmol/L)	4.853 (1.854)	5.152 (1.827)	4.566 (1.834)	<0.0001	0.3197
SUA (mean (SD) (umol/L)	324.783 (82.486)	362.709 (73.129)	288.397 (74.144)	<0.0001	1.0091
TC (mean (SD)) (mmol/L)	4.990 (1.040)	4.907 (1.026)	5.069 (1.046)	<0.0001	0.1557
LDL (mean (SD)) (mmol/L)	2.985 (0.914)	3.002 (0.919)	2.969 (0.909)	0.0563	0.0353
HDL (mean (SD)) (mmol/L)	1.392 (0.411)	1.254 (0.342)	1.524 (0.428)	<0.0001	0.6965
Smoking status, n (%)					
No	88909283.75 (54.07)	38140748.72 (47.38)	50768535.03 (60.50)	<0.0001	0.2656
Yes	75511631.21 (45.93)	42365705.51 (52.62)	33145925.70 (39.50)		
Drinking status, n (%)					
No	121735863.01 (74.04)	61966192.83 (76.97)	59769670.18 (71.23)	<0.0001	0.1314
Yes	42685051.95 (25.96)	18540261.39 (23.03)	24144790.55 (28.77)		
Hypertension, n (%)					
No	90832431.39 (55.24)	42286700.95 (52.53)	48545730.43 (57.85)	<0.0001	0.1072
Yes	73588483.57 (44.76)	38219753.28 (47.47)	35368730.29 (42.15)		
Hyperlipidemia, n (%)					
No	44906784.78 (27.31)	22834075.09 (28.36)	22072709.69 (26.30)	0.0397	0.0462
Yes	119514130.17 (72.69)	57672379.14 (71.64)	61841751.03 (73.70)		
DM, n (%)					
No	20590822.85 (12.52)	10839056.54 (13.46)	9751766.31 (11.62)	0.0024	0.0557
Yes	143830092.10 (87.48)	69667397.69 (86.54)	74162694.42 (88.38)		
HUA, n (%)					
No	134462655.02 (81.78)	63825587.55 (79.28)	70637067.47 (84.18)	<0.0001	0.127
Yes	29958259.94 (18.22)	16680866.68 (20.72)	13277393.26 (15.82)		
PIR (median [IQR])	3.090 [1.560, 5.000]	3.230 [1.670, 5.000]	2.920 [1.460, 4.970]	<0.0001	0.1104
TG (median [IQR]) (mmol/L)	1.150 [0.790, 1.680]	1.231 [0.847, 1.780]	1.080 [0.756, 1.581]	<0.0001	0.2235
insulin (median [IQR]) (pmol/L)	55.140[36.000, 88.440]	56.760[36.660, 91.730]	53.820 [35.280, 84.900]	0.0004	0.0862
albumin (median [IQR]) (mg/L)	7.500 [4.100, 14.500]	7.600 [4.300, 14.100]	7.400 [3.900, 14.900]	0.1264	0.0176
creatinine (median [IQR]) (umol/L)	10343.000[6100.000, 15205.000]	12287.600[7779.200, 17149.600]	8575.000[4950.400, 13260.000]	<0.0001	0.5031

Among the quantiles of the BUN, there were significant between-group differences in all baseline characteristics except for DBP, HDL and smoking status(*p*>0.05). Participants in the highest quantile.

of the BUN were more likely to be male, be Non-Hispanic White, be widowed/divorced/separated and be drinkers; had the highest age, weight, WC, BMI, height, SBP, HbA1c, FPG, BUN, SUA, TC, LDL, creatinine, insulin, TG, albumin and PIR; and had the highest prevalence of HUA, hypertension and hyperlipidemia (all *p* < 0.05). In addition, participants in the group with the lowest BUN were more likely to be non-Hispanic Black and never married; had the lowest proportion of smokers and the lowest prevalence of DM (*p <*0.01; **Table 1**).

Compared to the female group, male participants were typically younger, more likely to be Mexican American, more likely to be married/living with a partner or never married, less than high school education, smokers, hypertensive, and HUA; higher PIR, weight, WC, height, SBP, DBP, HbA1c, FPG, BUN, SUA,TG, insulin, and creatinine; lower BMI, TC, and HDL; and lower incidence of alcohol abuse, DM and hyperlipidemia (*p <*0.05). Nevertheless, both female and male populations had similar levels of LDL and albumin (all *p >*0.05; **Table 2**).

### Risk factors for HUA in the selected population

As shown in **Table 3**. By univariate analysis, we found that age, race, sex, marital status, education level, weight, WC, BMI, height, SBP, DBP, HbA1c, FPG, insulin, albumin, creatinine, BUN, TC, TG, LDL, HDL, DM status, smoking status, hypertension status and hyperlipidemia status were associated with the risk of developing HUA (all *p* < 0.05). However, PIR and drinking status were not related to HUA incidence (**Table 3**).

**Table 3:** Univariate analysis of the association between risk factors and HUA.

Variable	OR (95%CI)	*p* value
Age (years)	1.02 (1.02,1.02)	<0.001
Age group, n (%)		
20-44	1 (Ref)	
45-59	1.21 (1.09,1.34)	<0.001
60-74	1.90 (1.71,2.10)	<0.001
≥75	2.46 (2.17,2.79)	<0.001
Race, n (%)		
Mexican American	1 (Ref)	
Other Hispanic	1.07 (0.9,1.28)	0.43
Non-Hispanic White	1.6 (1.42,1.8)	<0.001
Non-Hispanic Black	1.9 (1.66,2.16)	<0.001
Other Race	1.52 (1.29,1.78)	<0.001
Gender, n (%)		
Male	1 (Ref)	
Female	0.76 (0.71,0.82)	<0.001
Marital status, n (%)		
Married/Living with Partner	1 (Ref)	
Widowed/Divorced/Separated	1.29 (1.19,1.41)	<0.001
Never married	0.87 (0.78,0.98)	0.018
Education level, n (%)		
Less Than High School	1 (Ref)	
High School Diploma (including GED)	1.2 (1.07,1.33)	0.001
More Than High School	1.01 (0.92,1.11)	0.778
PIR	1 (0.98,1.02)	0.936
Weight (kg)	1.03 (1.02,1.03)	<0.001
WC (cm)	1.04 (1.04,1.04)	<0.001
BMI(kg/m2)	1.09 (1.08,1.09)	<0.001
Height (cm)	1.01 (1.01,1.01)	<0.001
SBP (mmHg)	1.02 (1.01,1.02)	<0.001
DBP (mmHg)	1.01 (1.01,1.01)	<0.001
HbA1c (%)	1.13 (1.09,1.16)	<0.001
FPG, mmol/L	1.07 (1.05,1.09)	<0.001
Insulin, pmol/L	1 (1,1)	<0.001
Albumin, mg/L	1 (1,1)	<0.001
Creatinine, umol/L	1 (1,1)	<0.001
BUN, mmol/L	1.28 (1.26,1.3)	<0.001
BUN quantiles, (%)		
Q1	1 (Ref)	
Q2	1.41 (1.27,1.57)	<0.001
Q3	2.66 (2.41,2.94)	<0.001
TC, mmol/L	1.1 (1.06,1.14)	<0.001
TG, mmol/L	1.7 (1.62,1.78)	<0.001
LDL, mmol/L	1.09 (1.05,1.14)	<0.001
HDL, mmol/L	0.42 (0.38,0.47)	<0.001
DM n (%)		
No	1 (Ref)	
Yes	0.53 (0.48,0.58)	<0.001
Smoking status n (%)		
No	1 (Ref)	
Yes	1.22 (1.14,1.32)	<0.001
Drinking status n (%)		
No	1 (Ref)	
Yes	1.01 (0.93,1.1)	0.798
Hypertension n (%)		
No	1 (Ref)	
Yes	1.53 (1.42,1.65)	<0.001
Hyperlipidemia n (%)		
No	1 (Ref)	
Yes	2.14 (1.94,2.37)	<0.001

OR, odds ratio; CI, confidence interval; Ref, reference.

### Effect of the BUN on HUA incidence

The associations between BUN and HUA were analyzed using multivariate logistic regression and subgroup analyses stratified by sex, and the results are shown in **Table 4**. In the fully adjusted model (Model 4) adjusted for age, race, gender, marital status, education level, WC, BMI, insulin, albumin, hypertension status, DM status, smoking status, drinking status, hyperlipidemia status, the positive relationships between the BUN and the risk of HUA were found as a quantile variable, Q3 vs. Q1, OR (95%CI): 1.93(1.65~2.24), *p* < 0.05, *p* for trend < 0.001; as a continuous variable, per 1 mmol/L increment, OR (95%CI): 1.24(1.20~1.28), *p* < 0.001.Besides, among men, per 1 mmol/L increment in BUN, the risk of HUA increased by 18% [OR (95%CI): 1.18(1.13~1.24)] in the fully adjusted model. When the BUN was divided into three groups according to quantile, we compared the Q1 with the adjusted OR of Q2 and Q3, which were 1.19 (95% CI: 0.99~1.43) and 1.68 (95% CI: 1.38~2.04) in model 4. Among women, per 1 mmol/L in BUN, the risk of HUA increased by 31% [OR (95%CI): 1.31 (1.23~1.39)] in the fully adjusted model. The multivariable-adjusted OR for HUA compared Q1 with Q2 and Q3, which were 1 (95% CI: 0.74~1.37) and 2 (95% CI: 1.45~2.76). The highest level of BUN was associated with an increased risk of HUA.

**Table 4:** Multivariate analysis of the association between the BUN and the risk of developing HUA.

Variable	Model 1		Model 2		Model 3		Model 4	
	OR (95%CI)	*p* value	OR (95%CI)	*p* value	OR (95%CI)	*p* value	OR (95%CI)	*p* value
Total (n=17846)								
BUN	1.26 (1.23,1.29)	<0.001	1.22 (1.19,1.26)	<0.001	1.23 (1.19,1.27)	<0.001	1.24 (1.20,1.28)	<0.001
BUN quantiles								
Q1 (n=5076)	1 (Ref)		1 (Ref)		1 (Ref)		1 (Ref)	
Q2 (n=6393)	1.32 (1.17,1.49)	<0.001	1.23 (1.08,1.39)	0.001	1.18 (1.03,1.36)	0.015	1.2 (1.05,1.37)	0.009
Q3 (n=6377)	2.28 (2.01,2.59)	<0.001	1.92 (1.66,2.21)	<0.001	1.89 (1.62,2.21)	<0.001	1.93 (1.65,2.24)	<0.001
Trend test		<0.001		<0.001		<0.001		<0.001
Male (n=8880)								
BUN	1.14 (1.10,1.18)	<0.001	1.16 (1.11,1.20)	<0.001	1.16 (1.11,1.21)	<0.001	1.18 (1.13,1.24)	<0.001
BUN quantiles								
Q1 (n=1863)	1 (Ref)		1 (Ref)		1 (Ref)		1 (Ref)	
Q2 (n=3265)	1.2 (1.00,1.44)	0.045	1.21 (1.01,1.45)	0.043	1.17 (0.97,1.40)	0.1	1.19 (0.99,1.43)	0.071
Q3 (n=3752)	1.58 (1.33,1.87)	<0.001	1.61 (1.33,1.95)	<0.001	1.61 (1.33,1.95)	<0.001	1.68 (1.38,2.04)	<0.001
Trend test		<0.001		<0.001		<0.001		<0.001
Female (n=8966)								
BUN	1.39 (1.33,1.45)	<0.001	1.29 (1.23,1.36)	<0.001	1.3 (1.22,1.37)	<0.001	1.31 (1.23,1.39)	<0.001
BUN quantiles								
Q1 (n=3213)	1 (Ref)		1 (Ref)		1 (Ref)		1 (Ref)	
Q2 (n=3128)	1.23 (0.98,1.54)	0.066	1.07 (0.84,1.37)	0.548	0.99 (0.76,1.29)	0.924	1 (0.74,1.37)	0.973
Q3 (n=2625)	3 (2.44,3.69)	<0.001	2.05 (1.60,2.63)	<0.001	1.95 (1.48,2.58)	<0.001	2 (1.45,2.76)	0.003
Trend test		<0.001		<0.001		<0.001		0.001

For total population: Model 1: unadjusted; Model 2: age, race, gender, marital status and education level; Model 3, adjusted for age, race, gender, marital status, education level, WC, BMI, insulin, albumin and creatinine; Model 4, adjusted for age, race, gender, marital status, education level, WC, BMI, insulin, albumin, creatinine, hypertension status, DM status, smoking status, drinking status and hyperlipidemia status. For male/female population: Model 1~4: adjusted for the same covariates as above, except for the gender characteristics used for stratification.

### Subgroup analyses

Stratified subgroup analysis was conducted in the study population to evaluate whether the relationship between BUN and HUA remained consistent or differed across different demographic characteristics and disease status groups.

**Figure 2:**
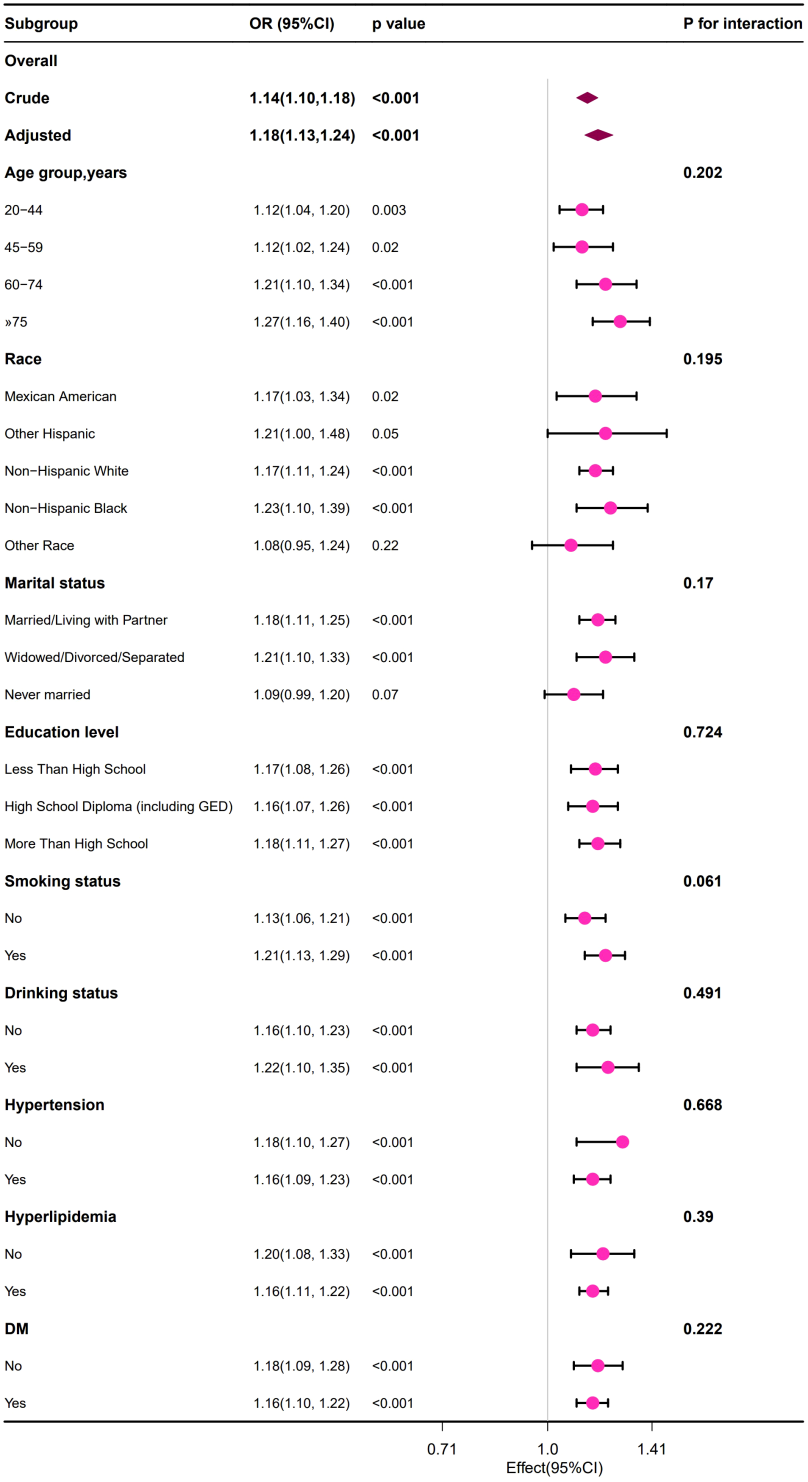
Forest plot of the association between the BUN and the risk of developing HUA in male group.

**Figure 3:**
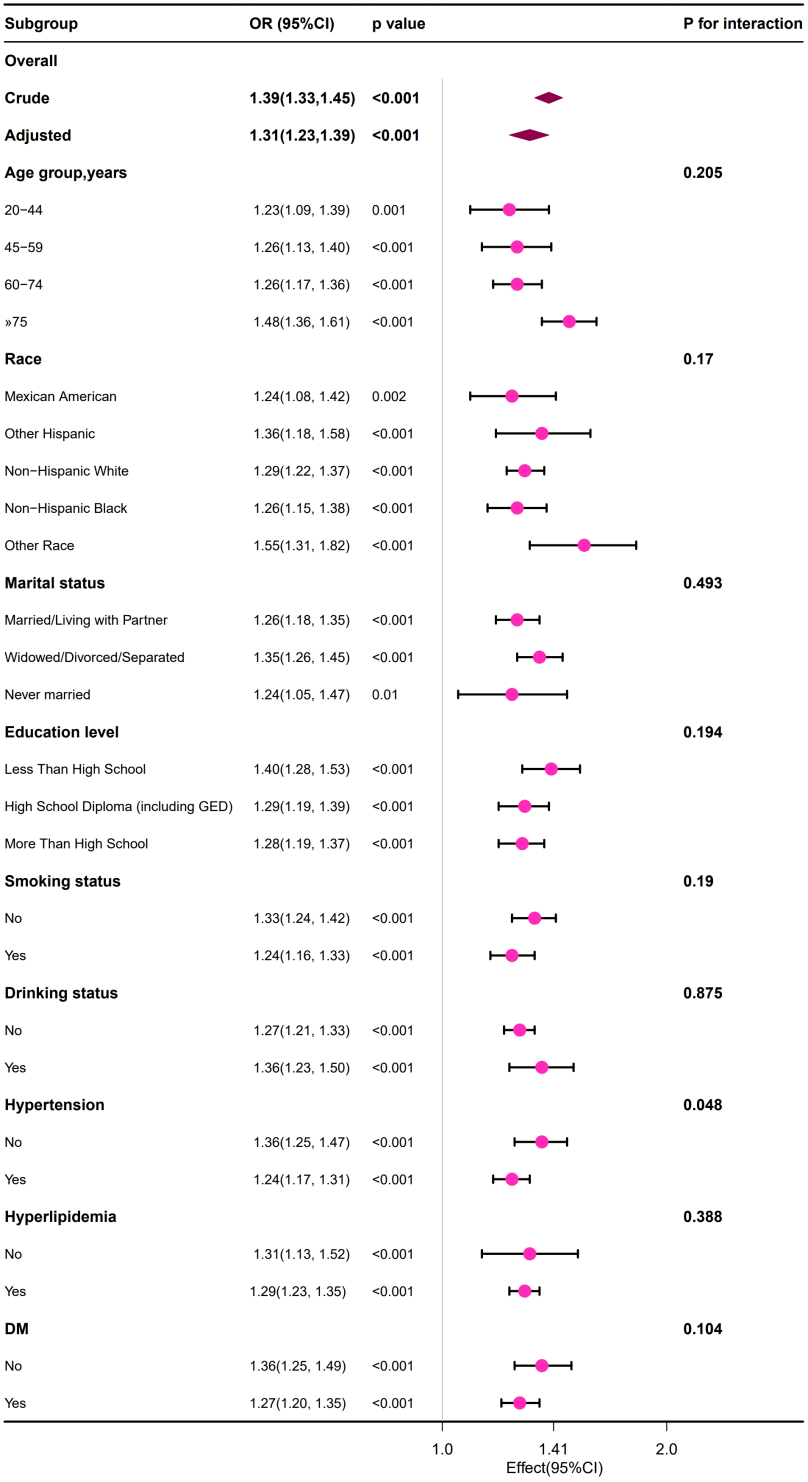
Forest plot of the association between the BUN and the risk of developing HUA in female group.

We observed that the positive relationship between the BUN and the risk of developing HUA remained consistent across all subgroup variables in male and female population (**Figures 2**, **3**). Interestingly, we also found an interaction effect in the female subgroup, whereby the association between BUN and HUA was stronger in the non-hypertensive group (OR=1.36, 95% CI: 1.25 ~1.47) (*p*=0.048) (**Figure 3**).

### Interaction of sex with the association between BUN and HUA

**Figure 4:**
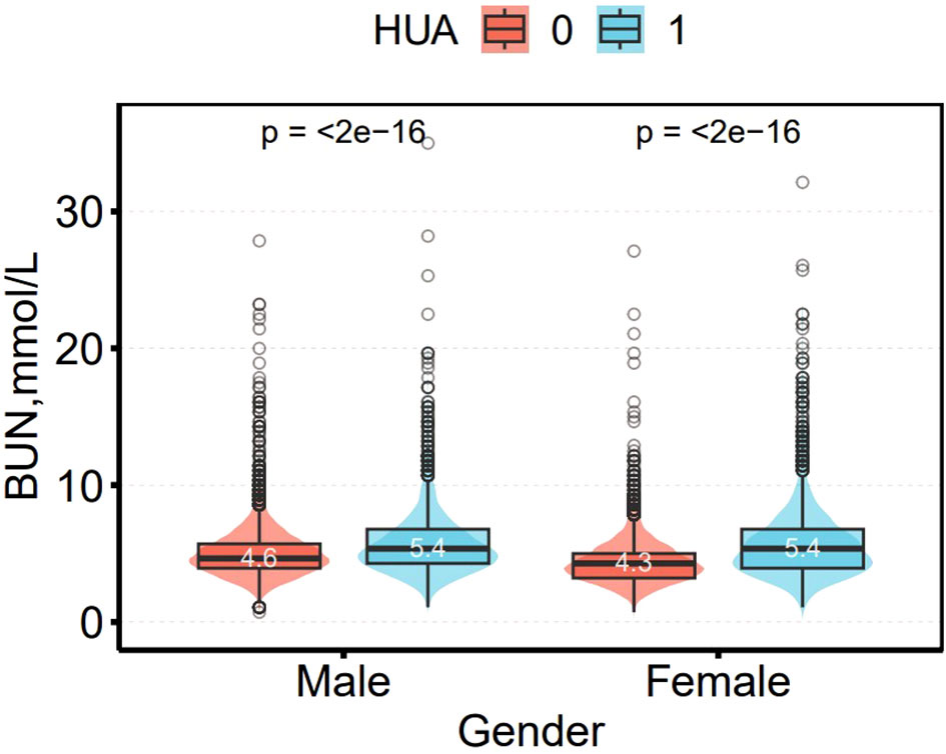
Distribution of BUN in the men and women groups.

**Figure 4** shows the difference in BUN between non-HUA and HUA participants. Among male and female participants, BUN levels were significantly higher in the HUA group than in the non-HUA group, (4.6 vs. 5.4 mmol/L) and (4.3 vs. 5.4 mmol/L (all *p* < 0.01), respectively.

After adjusting for age, race, gender, marital status, education level, WC, BMI, insulin, albumin, hypertension status, DM status, smoking status, drinking status, hyperlipidemia status, as BUN increased, the risk of HUA increased significantly in both men (OR: 1.19, 95% Cl:1.16~1.23) and women (OR:1.32, 95% Cl:1.32 (1.28~1.37)(all *p* < 0.001). The interaction between gender status and the prevalence of BUN and HUA was significant (*p* for interaction likelihood ratio test <0.01). This interaction remained significant when converting BUN to categorical variables (*p* for interaction likelihood ratio test <0.01) (**Table 5** **).**

**Table 5:** Interactive effect of BUN and HUA in men and women groups.

Variable	Male (n=8880)		Female (n=8996)		*p* for interaction
	OR (95%CI)	*p* value	OR (95%CI)	*p* value	
BUN, mmol/L	1.19 (1.16,1.23)	<0.001	1.32 (1.28,1.37)	<0.001	<0.001
BUN quantiles, n (%)					<0.001
Q1	1(Ref)		1(Ref)		
Q2	1.2 (1.03,1.41)	0.022	1.21 (1.02,1.43)	0.026	
Q3	1.8 (1.53,2.11)	<0.001	2.59 (2.18,3.07)	<0.001	
Trend.test	1.37 (1.26,1.48)	<0.001	1.66 (1.52,1.81)	<0.001	

### The nonlinear relationship between BUN and HUA

**Figure 5:**
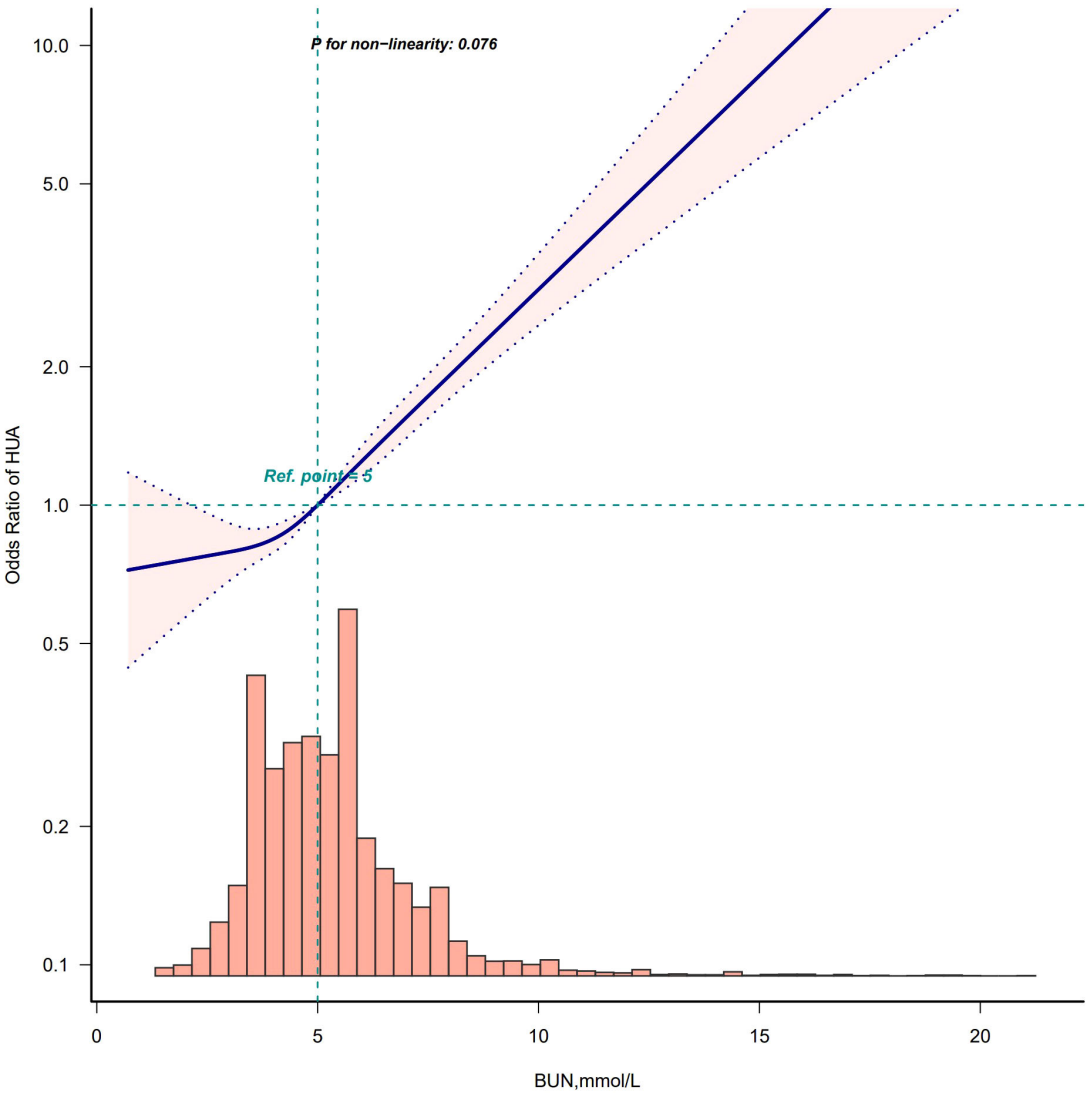
Restricted cubic spline model of the odds ratios of BUN with HUA in male. Adjusted for Model 4: age, race, marry status, education level, WC, BMI, insulin, albumin, creatinine, hypertension, DM, smoke, drink, hyperlipidemia.

**Figure 6:**
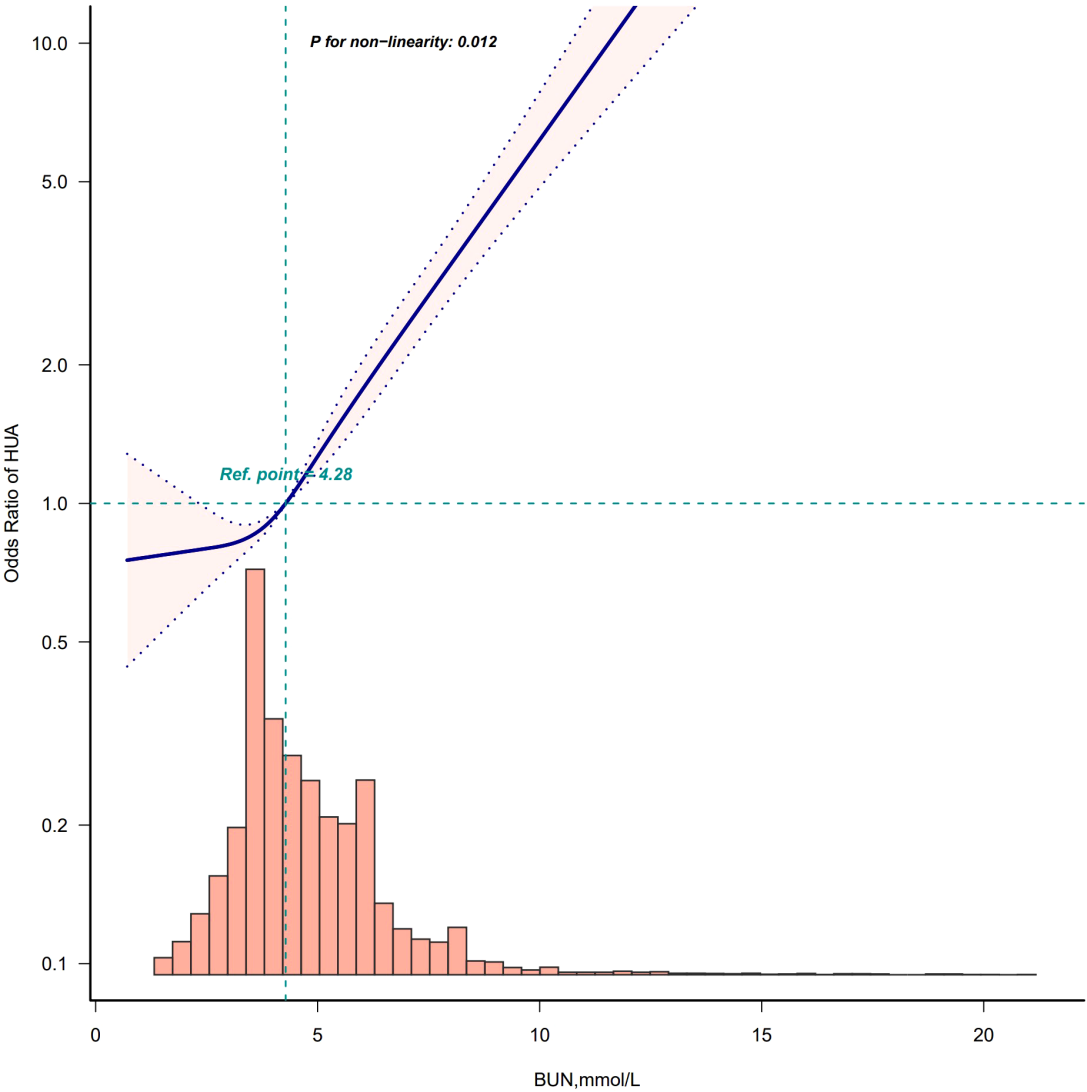
Restricted cubic spline model of the odds ratios of BUN with HUA in female. Adjusted for Model 4: age, race, marry status, education level, WC, BMI, insulin, albumin, creatinine, hypertension, DM, smoke, drink, hyperlipidemia.

After adjusting according to Model 4, the fitting curves of the BUN and the risk of developing HUA were drawn to better explain their relationships. The results showed that there was a straightforward linear relationship between the BUN and the risk of developing HUA in males (*p* for nonlinearity = 0.076) (**Figure 5**), while the dose-response relationship between the BUN and HUA in females was positive in a nonlinear manner (*p* for nonlinearity = 0.012) (**Figure 6**).

## Discussion

In this extensive population-based cross-sectional study, we found that BUN was significantly and positively associated with HUA in U.S. adults, after full adjustment for all potential confounders. Additional assessment of gender differences between BUN and HUA prevalence showed a stronger correlation in female subjects and a nonlinear correlation in females. To our knowledge, this is the first study describing the association of BUN with the development of HUA in the general population of the United States and gender differences. These findings have important clinical implications for HUA management strategies in Western populations, particularly given the escalating HUA prevalence and associated multi-organ morbidity.

Previous studies have shown that BUN is strongly associated with a variety of metabolic diseases, such as DM (22, 31, 32), diabetic retinopathy (33), hyperlipidemia (23) and fatty liver (25). However, few studies have evaluated the relationship between BUN and the risk of HUA development in the general adult population, particularly in the United States. In this study, we used data from NHANES, which, after weighted analysis, found that BUN was positively associated with HUA in U.S. adults (OR,1.24; 95%CI: 1.20-1.28) after adjusting for age, race, sex, marital status, education level, BMI, insulin, albumin, hypertensive status, DM status, smoking status, drinking status, and hyperlipidemia status. Besides, in this study we performed curve-fitting analysis and subgroup analysis to further confirm the correlation between BUN and HUA. In this study, we used data from NHANES, which, after weighted analysis, found that BUN was positively associated with HUA in U.S. adults (OR,1.24; 95%CI: 1.20-1.28) after adjusting for age, race, sex, marital status, education level, BMI, insulin, albumin, hypertensive status, DM status, smoking status, drinking status, and hyperlipidemia status. Besides, in this study we performed curve-fitting analysis and subgroup analysis to further confirm the correlation between BUN and HUA.

To the best of our knowledge, only one cross-sectional study in China has shown that BUN is a risk factor for HUA (34). The study showed that among low-income adults in rural North China, high BUN levels were significantly associated with a higher risk of developing HUA. The results were consistent with our findings. However, the study involved fewer logistic regression-adjusted variable models, a small sample size, no further subgroup or curve-fitting analyses, and a study population of middle-aged and older adults (aged >50 years) in rural northern China, which limited its external validity. With a focus on the US population, our study may complement the findings of previous research. Because we used an NHANES design to obtain estimates that were nationally representative of the US, our results should be generalizable to adults in the United States.

The biological mechanisms underlying the association between the BUN and the risk of HUA have not been elucidated, with some of the following potential explanations. Elevated BUN: (i) usually reflects abnormal kidney function, which may include chronic kidney disease or acute kidney injury. When kidney function is impaired, the ability to excrete urea and uric acid may be reduced, resulting in higher concentrations of urea and uric acid in the blood (35); (ii) associated with a high-protein diet. High-protein diets may lead to elevated BUN and impaired renal function (36–38), and also increase uric acid production because uric acid is metabolized from purines, and high-protein foods are usually high in purines; (iii)associated with increased insulin resistance. Researchers have now found that urea can directly act on pancreatic β-cells, thus affecting insulin secretion and inducing insulin resistance through various mechanisms such as oxidative stress and inducing post-translational modifications (e.g., O-GlcNAcylation) (39), and insulin resistance is closely related to HUA (40–43); (iv)related to insufficient renal tubular reabsorption. Elevated BUN may lead to impaired renal tubular function, which affects excretion and reabsorption of uric acid, leading to elevated uric acid concentration in the blood. These mechanisms may lead to increased production or decreased excretion of uric acid in the body, ultimately leading to the development of HUA.

In this study, the overall prevalence of HUA in U.S. adults from 1999 to 2020 was 18.22%, with 20.72% in men and 15.82% in women. Our study was in general agreement with the findings of Che et al., who showed that the overall prevalence of HUA in the United States from 2007–2018 was 19.0% (5841/30819), with prevalence rates of 21.04% (3179/15112) and 16.95% (2662/15707) in men and women, respectively (12). However, Singh et al. found that the overall prevalence of HUA in U.S. adults was 14.6% from 2007–08 to 2015–16 (1) and Chen-Xu et al. found that the prevalence of HUA in adult men and women in the United States in 2015–2016 was 20.2% and 20.0%, respectively (10). The results of these two U.S. population studies was somewhat different from ours, and possible reasons for this were considered to be that the diagnostic criteria for HUA were not the same as the year of the study population. In our and the other U.S. population studies mentioned above, the sex differences in the prevalence of HUA were not very dramatic. However, this is not the case in East Asian countries. Zhang et al. found that the prevalence of HUA in Chinese adult males and females was 19.3% and 2.8% respectively in 2015-2016, while in 2018–2019 the prevalence was 24.4% in males and 3.6% in females (11). Pia et al. found that in 2015–2017 prevalence of HUA in Chinese adult males and females was 21.2% and 8.5%, respectively (44). Kawano et al. showed that the prevalence of HUA in Japanese adult males and females was 4.87% (551/11324) and 0.78% (146/18683), respectively (45). Koo et al. found that the prevalence of HUA in Korean adult males and females was 13.33% and 0.82%, respectively (46). Sex differences in the prevalence of HUA are more pronounced in East Asian countries, possibly due to marked differences in lifestyle and dietary habits between men and women within these countries, and genetic factors may also be a contributing factor.

The present study further investigated gender differences in the correlation between BUN and HUA and found that the correlation between BUN and HUA may be stronger in females than in males, and that the correlation between BUN and HUA in females was nonlinear, whereas the correlation between BUN and HUA in males was linear. Furthermore, in the female group only, the correlation was even higher in non-hypertensive than in hypertensive patients. These gender differences may primarily involve three mechanisms: (i) sex hormones regulating uric acid metabolism, (ii) renal clearance disparities, and (iii) lifestyle factors’ varied impacts. Sex hormone regulation may play a pivotal role. Experimental studies showed that estrogen enhances renal urate excretion through downregulation of urate transporter 1 and glucose transporter 9 transporters, while testosterone upregulated these reabsorption channels (47–50). This hormonal dichotomy may explain why premenopausal women exhibit lower SUA levels than age-matched males. Interestingly, the nonlinear correlation pattern in females may reflect the dynamic estrogen fluctuations during menstrual cycles and menopausal transition. The results of the present study corroborated with previous studies: Li et al. (51) found a nonlinear dose-response relationship between estrogen levels and the risk of HUA in women, whereas Cho et al. (52) reported a significant increase in the prevalence of HUA from the late menopausal transition stage, which was highly compatible with the gender-differentiated features observed in the present study. Renal clearance disparities warrant particular attention. Although males generally have higher glomerular filtration rates, gender-specific urea handling mechanisms may exist. An animal study confirmed the presence of the Urea Transporter B2 in the rumen of deer rumen and significantly higher levels of Urea Transporter B2 in adult females than in adult males, suggesting a potential pathway for gender-specific urea-urate regulation (53). Furthermore, estrogen’s modulatory effects on the renin-angiotensin-aldosterone system could create sex-dimorphic renal hemodynamic environments affecting both BUN and SUA homeostasis. Lifestyle factors also show significant gender differences, broadly defined to include dietary patterns, physical activity, and psychological and social behaviors. Liu et al. found that for men, dietary habits had a greater impact on the likelihood of developing HUA, whereas for women, HUA was more associated with physical activity-related lifestyle choices (e.g., type of work, commute, and exercise) (54). Feraco et al. showed that men preferred to consume meat and to participate in strength and endurance sports, whereas women consumed more vegetables and were less physically active than men (55). New evidence suggests that these sex-specific lifestyle differences may arise from biological mechanisms (e.g., metabolic differences regulated by sex hormones) (56) and evolutionary adaptations shaped by energy allocation strategies and reproductive priorities (57). Although the exact mechanisms are unknown and further research is needed, gender factors need to be emphasized in the early management of HUA.

BUN has the advantages of easy and inexpensive detection and has the basic conditions to be used as a large-scale screening marker, but its level is easily affected by a variety of factors such as protein intake, dehydration, renal blood flow, etc., and its accuracy is not high and its application alone has some limitations. Our findings reveal positive correlation between BUN levels and HUA risk, indicating that BUN might be used as a risk stratification tool in clinical practice. However, the current study provides only preliminary evidence of correlation and a comprehensive risk stratification model has not yet been developed; more data need to be collected at a later stage and risk assessment models need to be constructed and validated in combination with other risk factors.

This study has several strengths: (i) it explored the relationship between BUN and HUA in the general U.S. population with gender-stratified analysis;(ii) the data for this study were obtained from a national population-based prospective survey, providing a representative sample of the population and increased the credibility of the study; (iii) the data were subjected to weighted statistical analysis, which enabled us to generalize the results to a larger sample size of the population. Nevertheless, there are some limitations in this study: (i) due to the cross-sectional design of NHANES, the temporal relationship between exposure and outcome cannot be established, limiting our ability to infer causality; thus, the association between BUN and the risk of developing HUA needs to be clarified by further cohort studies; (ii) although we adjusted for a variety of confounders in the multiple regression analysis, other potential confounders may still exist. Despite these limitations, these data effectively examine the relationship between BUN and the development of HUA, providing additional evidence on this topic and revealing possible sex differences in the association.

## Conclusion

The current study shows that elevated BUN is proportionally associated with an increased risk of developing HUA in the general adult population in the United States, with gender differences. These results suggest that BUN may be an independent risk factor for HUA, and possible gender differences warrant attention in the management of HUA prevention. However, further prospective studies are needed to elucidate the causality and potential mechanisms of the above associations to break through the limitation that cross-sectional studies cannot determine causality.

## Data Availability

Publicly available datasets were analyzed in this study. This data can be found here: https://www.cdc.gov/nchs/nhanes/index.htm.
